# Influence of Thermal Exposure on the Microstructure and Fatigue Performance of Laser Shock Peened Ti6Al4V Alloy

**DOI:** 10.3390/ma19204387

**Published:** 2026-10-19

**Authors:** Wentai Ouyang, Li Zhang, Zhaolin Wang, Wenwu Zhang, Liyuan Sheng

**Affiliations:** 1 Zhejiang Key Laboratory of Laser Extreme Manufacturing for Difficult-to-Machine Materials, Ningbo Institute of Materials Technology and Engineering, CAS, Ningbo 315201, China; 2 Avic Harbin Aircraft Industry Group Co., Ltd., Harbin 150060, China; 3 Shenzhen Institute, Peking University, Shenzhen 518057, China; 4 PKU-HKUST Shenzhen-Hongkong Institution, Shenzhen 518057, China

**Keywords:** laser shock peening, thermal exposure, Ti6Al4V alloy, microstructure, fatigue performance

## Abstract

In the present study, the influence of thermal exposure on the microstructural evolution and fatigue performance of LSP-treated Ti6Al4V alloy was systematically investigated in order to demonstrate the probable variation for the LSP-strengthened Ti6Al4V alloy components in temperature-rise environment, particularly for those components that had to be strengthened wholly. For this research target, the double-side surfaces were strengthened, while the side walls retained their original state, and a relatively high-temperature environment with 600 °C/1 h was applied on the LSP-treated samples to accelerate the influence of thermal environment. The results reveal that LSP reconstructs the superficial layer by refining coarse grains and merging ultrafine grains into larger ones while introducing high-density dislocations and {10–12} twins. These microstructural changes reduce the grain size difference and enhance the resistance to crack initiation and propagation, leading to a fatigue life 3.33 times that of the as-received (AR) state. After thermal exposure, the outermost nanograins and twins are preserved. Meanwhile, the difference in grain size is further decreased, accompanying the relatively rational distribution of different-sizes grains, which further decreases the fatigue striation width in the superficial layer. However, the matrix and adjacent regions undergo significant microstructural degradation, including grain coarsening, dislocation recovery and unsuitable dislocation redistribution, which promote multiple crack initiation sites and decrease the fatigue life to only 10.5% higher than the AR state. These findings provide valuable insights into the service reliability of LSP-strengthened titanium alloy components operating under thermal–mechanical coupling conditions.

## 1. Introduction

Owing to the well-balanced servicing performances, such as exceptional specific strength and favorable corrosion resistance, the Ti-6Al-4V alloy has been extensively utilized in the turbocharged critical components with integrated manufacturing [1,2]. However, during service, the turbocharged components are exposed to severe operating conditions, typically subjected to high-cycle fatigue loading, cyclic high-temperature environment, and foreign object damage. Such combined actions readily weaken microstructure and induce surface crack initiation, leading to a significant reduction in component lifetime [3,4]. Given that fatigue failure predominantly originates from the surface or near-surface regions, surface strengthening techniques are indispensable for improving the service reliability and extending the operational life of these critical components.

Laser shock peening (LSP) has emerged as a highly effective surface severe plastic deformation technique, which is capable of significantly retarding crack initiation and propagation through the generation of high-magnitude compressive residual stresses, the introduction of high-density dislocations, and the refinement of near-surface grain structures [5,6]. These synergistic effects contribute substantially to enhancing resistance against fatigue, wear, and impact damage [7,8]. Recent studies have consistently demonstrated that LSP markedly improves the fatigue strength and fatigue life of Ti-6Al-4V alloy at ambient temperatures [9]. The formation of a hybrid strengthening layer, comprising both crystal defects and refined grains, plays a pivotal role in achieving such performance enhancement [10,11]. Moreover, the beneficial effects of LSP on wear resistance and foreign object damage tolerance have also been well documented [12]. These advantages collectively establish LSP as a promising surface strengthening strategy for turbocharged components operating under demanding service conditions.

Despite the well-documented benefits of LSP at room temperature, turbocharged components frequently operate at elevated temperatures. The working temperature of Ti6Al4V alloy is generally limited to approximately 350 °C due to its limited resistance to creep and oxidation behaviors [13]. At such temperatures, the microstructural stability and thermal stability of the beneficial effects induced by LSP, including compressive residual stresses and dislocation structures, become critical concerns [14,15]. Especially for some specific conditions, the environment temperature could even exceed such levels and lead to detrimental effects on components. Some previous researchers have found this problem and investigated the stability of LSP-treated alloys in elevated temperatures. Zhou et al. [16] investigated the relaxation of residual stress for the LSP-treated Ti6Al4V alloy within the temperature between 400 °C and 600 °C via both simulation and experiment and reported the severe relaxation on the surface and minor influence on the depth of residual compressive stress. Higher temperature would lead to a larger decrease in superficial compress stress, but the overall stability is great. Chin et al. [17] compared the thermal stability on residual compressive stress induced by LSP and shot peening (SP) within the temperature between 250 °C and 600 °C, based on Inconel 718 alloy, and found the deeper influencing depth and higher stability of LSP. Similarly, some other research also reported the preferably stable residual stress induced by LSP on Ni-based alloys [18,19], but the systematic research on the coupled evolution of its microstructure and fatigue performance under thermal exposure remains scarce, especially for titanium alloys. A comprehensive understanding of how thermal exposure influences the LSP-induced microstructure and, consequently, fatigue behavior is urgently needed to guide the safe and reliable application of LSP-strengthened components in high-temperature environments. Moreover, since the integrated manufacturing of turbocharged component, some regions could be partly processed, such as connecting sites. The influence of thermal environment on the side wall and matrix could significantly affect the fatigue performance. It is critical to evaluate the strengthening effect of LSP for this kind of structure.

In this study, the effects of thermal exposure on the microstructural evolution and fatigue performance of LSP-treated Ti6Al4V alloy were systematically researched, where the double-side surfaces were strengthened, but the side walls kept the original state. Specifically, the high temperature environment at 600 °C for 1 h was applied on LSP-treated specimens to accelerate the thermal influence procedure. Meanwhile, such a high temperature is meaningful for revealing the evolution of LSP-treated components in extremely harsh environments. The resulting changes in grain structure, dislocation distribution, and fatigue properties were characterized in detail. The findings could partly complement the deficiency of the evolution mode for LSP-treated Ti6Al4V alloy in high-temperature environment and are expected to provide valuable insights into the service reliability of LSP-strengthened titanium alloy components operating under thermal–mechanical coupling conditions.

## 2. Experimental Procedure

### 2.1. Sample Preparation

The commercially forged Ti6Al4V titanium alloys (Shanghai Gangda Supply Chain Co., Ltd., China) were used in this study. The dimension of as-received (AR) plate is about 120 mm × 120 mm × 15 mm, which was cut into small ones with the size of 120 mm × 120 mm × 3 mm by wire-cut electrical discharge (WED, SL400Q, Sodick, Japan) for the application of LSP. For preventing the influences of WED and surface contaminants, the surface of each plate was polished by sandpaper (400# and 1000#) and cleaned with ethanol before LSP processing, which was subsequently dried by cold wind.

**Figure 1:**
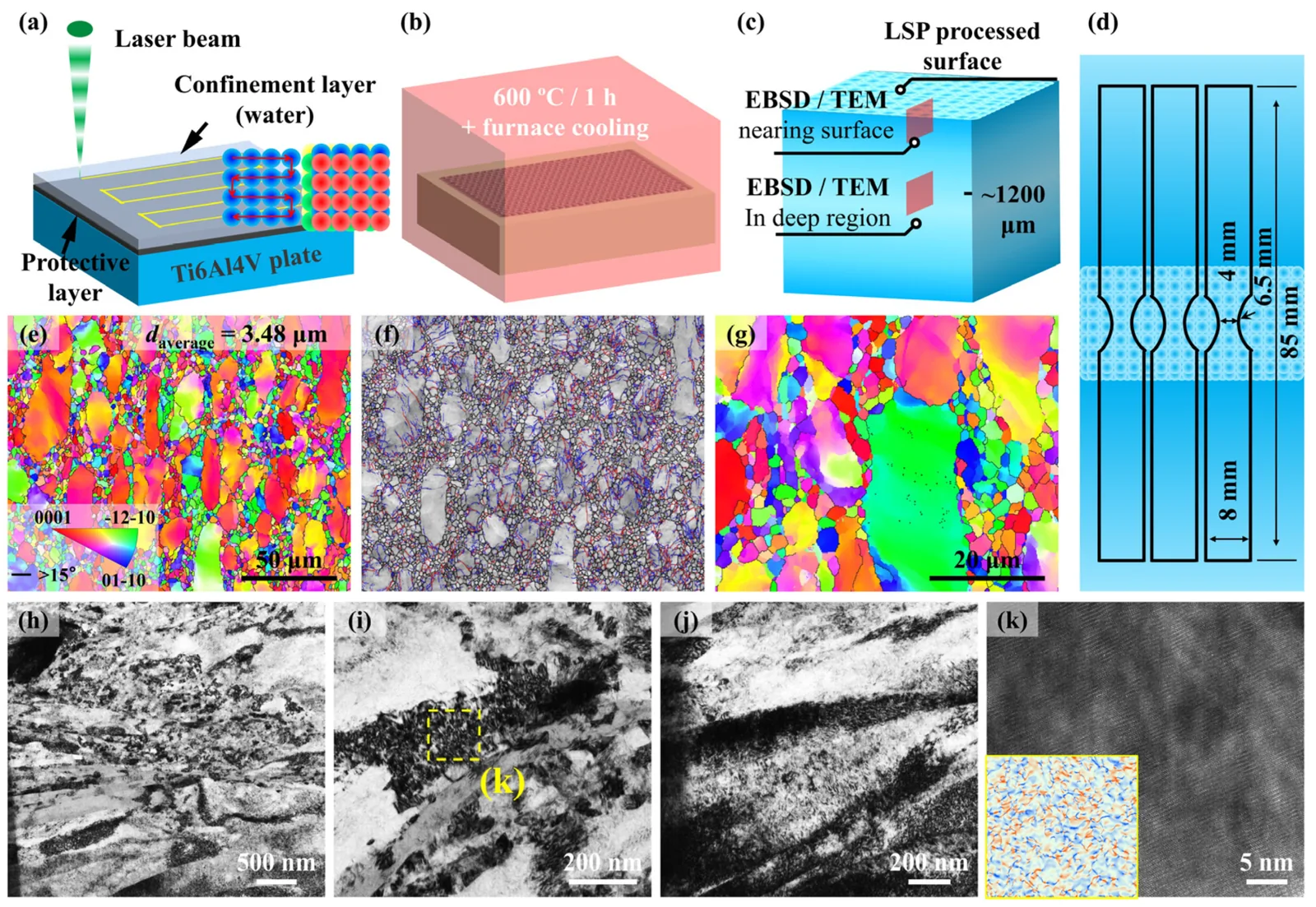
(**a**) Schematic diagram showing the LSP process; (**b**) Schematic diagram showing the thermal exposure; (**c**) Schematic diagram showing the sampling sites for EBSD and TEM observations; (**d**) Schematic diagram showing the dimensions of fatigue test sample; EBSD observations on the superficial layer of AR state: (**e**) Crystallographic orientation map with low magnification and its GB map covering on band contrast map (**f**), (**g**) Crystallographic orientation map with high magnification; TEM observations on the outermost region of AR state: (**h**–**j**) Bright-field image showing the grain structure and dislocation morphology, (**k**) HRTEM image showing the atomic structure in coarser grain (Inset image showing the corresponding ε_xy_ GPA strain image).

Figure 1e–k demonstrate the typical microstructure in superficial layer of forged Ti6Al4V alloy. As shown in Figure 1e, the original microstructure exhibits a distinct dual-size grain structure. The coarse grains mainly exhibit columnar morphology, and their length mostly exceed 20 μm, while the ultrafine grains mainly demonstrate the equiaxial morphology, accompanying with the size around 3 μm. Moreover, numerous low-angle grain boundaries (LAGBs) distribute in the edge region of coarse grains, indicating the localized distribution of high-density geometrically necessary dislocations (GNDs) [20]. Figure 1h–k display the crystal structures within the outermost region of forged Ti6Al4V alloy. It can be found that the region adjacent to surface also reveals the dual-size grain feature, and the coarser grains normally possess high-density dislocations as well. Such a microstructure state could provide considerable resistance to deformation in the initial stage, but the high dislocation density in coarse grains would weaken the strain accommodation capability, which could be adverse to resist the high-cycle fatigue behavior, especially for the region nearing grain boundary (GB) with excessive dislocation accumulation [21].

### 2.2. Laser Shock Peening and Thermal Exposure Process

A commercial Nd:YAG pulsed laser (SGR-60, Beamtech Optronics Co., Ltd., China) was applied to conduct LSP processing on Ti6Al4V plates with 10 Hz frequency, 14 ns pulse width, and 532 nm wavelength. A uniform water film with a thickness of about 2 mm was used as the confinement layer. In order to prevent thermal damage of pulsed laser on the sample, the black tape with a thickness of about 0.12 mm was stuck on the sample surface, which was frequently replaced to keep the insulation effect. A robotic arm (KR60, KUKA, China) was used to clamp the Ti6Al4V plate and conduct LSP treatment. After carefully screening processing parameters, the optimal LSP condition was selected as follows: laser beam diameter of 2 mm, laser energy density of 6.37 GW/cm^2^, and overlap ratio of 50% (both of X and Y directions). Accordingly, four-pass sectional laser processing was conducted on the sample surface to complete a one-time LSP treatment with an overlap ratio of 50%. Moreover, the two-layers LSP processing was used in this research based on balanced performance and efficiency.

After LSP processing, a tubular heat treatment furnace (1200C, Bayiker Equipment Technology Co., China) was applied to conduct thermal exposure. The thermal exposure was performed in the furnace with an air condition at 600 °C for 1 h. During the thermal exposure procedure, the Ti6Al4V plates were heated with a heating rate of 10 °C/min and cooled down with the furnace. For convenient identification, the two-layer LSP-processed samples were named as LSP in this research, while those samples subsequently treated by two-layer LSP and thermal exposure were named as LSP-TE.

### 2.3. Microstructural Characterization

Specimens for surface morphology observation and cross-sectional microstructure analysis were cut from the LSP-treated plates by WED. Subsequently, these specimens were ultrasonically cleaned in ethanol for 5 min to remove surface contaminants. Surface topography was observed using a laser scanning confocal microscope (LSCM, VK-X200, Keyence, Japan). The specimens for surface morphology observation and microstructure characterization were prepared by the conventional metallographic method, which embedded the specimens by cold mounting material, and then ground them by silicon carbide sandpapers from 500# to 2000# before polishing. The polishing was performed by felted cloth cooperating with the diamond polishing suspension liquid. Surface morphology and cross-sectional microstructure were characterized using a scanning electron microscope (SEM, Verios G4 UC, ThermoFisher, USA) equipped with energy-dispersive spectroscopy (EDS) and electron backscatter diffraction (EBSD) detectors. EBSD specimens were prepared by ion milling, while transmission electron microscope (TEM, Talos F200x, ThermoFisher, America) specimens were lifted out from the top surface of the LSP-treated plates using a focused ion beam (FIB, Auriga, ZEISS, Germany) system. TEM observations were performed at 200 kV with a resolution of 0.12 nm.

### 2.4. Fatigue and Surface Hardness Test

**Figure 2:**
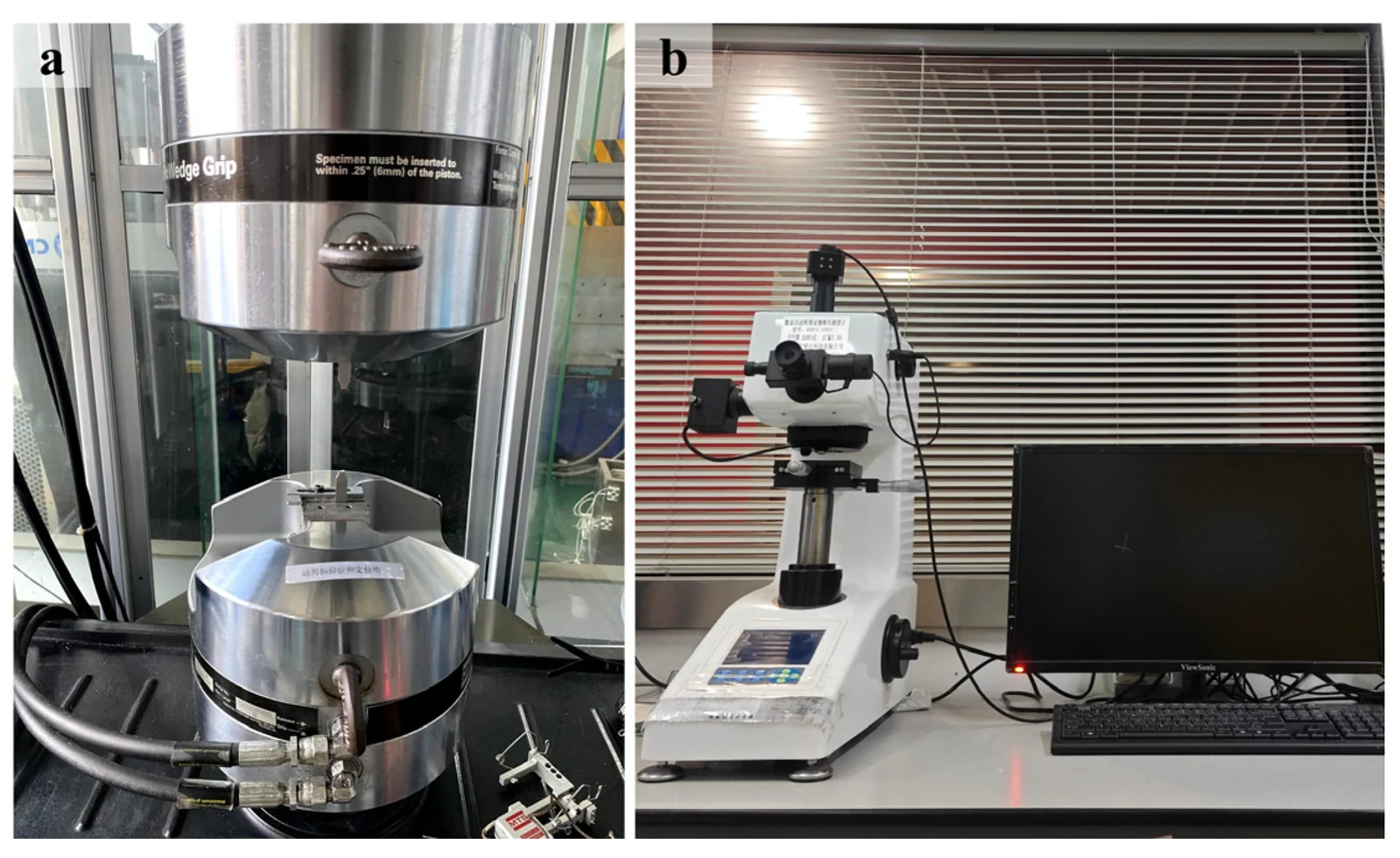
Physical image showing the (**a**) fatigue test equipment and (**b**) microhardness test equipment.

As shown in Figure 2a, the fatigue tests were conducted on the Landmark fatigue tester (MTS, USA) with the peak stress of 540 MPa and stress ratio of 0.1. Meanwhile, the loading waveform is the sine wave, and the frequency is 10 Hz. Figure 1d shows the specific size of fatigue test samples, meeting the requirements of standard. The fatigue samples were also cut by WED, and the surfaces and sidewalls were carefully ground and polished to remove the influence layer of WED, which demonstrate the roughness about Ra 0.5 μm. After careful grinding, the LSP and TE treatments were performed on corresponding samples, and no additional processing was conducted on surfaces and sidewalls. Moreover, the light blue zone in Figure 1d represents the LSP-treated region, which is much larger than the testing section and keeps consistent for each specimen. Three fatigue tests were performed for each condition to obtain an average fatigue life value. Moreover, a right angle was used before the fatigue test to ensure the coaxial state between sample and clamping. After testing, the regions nearing fatigue fracture were cut by WED to observe the fracture surface morphology via SEM. As shown in Figure 2b, the surface hardness test (RDHVS-1000Z Vickers, Beijing Shidai Ruida Technology Co., Ltd., China) was performed on the surface of different samples with the load and dwelling time of 25 g and 15 s, respectively, and nine positions were chosen for obtaining the average value. Both of the fatigue and hardness tests were performed in room temperature.

## 3. Results and Discussion

### 3.1. Evolution of Superficial Microstructure and Outermost Crystal Defects Induced by LSP

**Figure 3:**
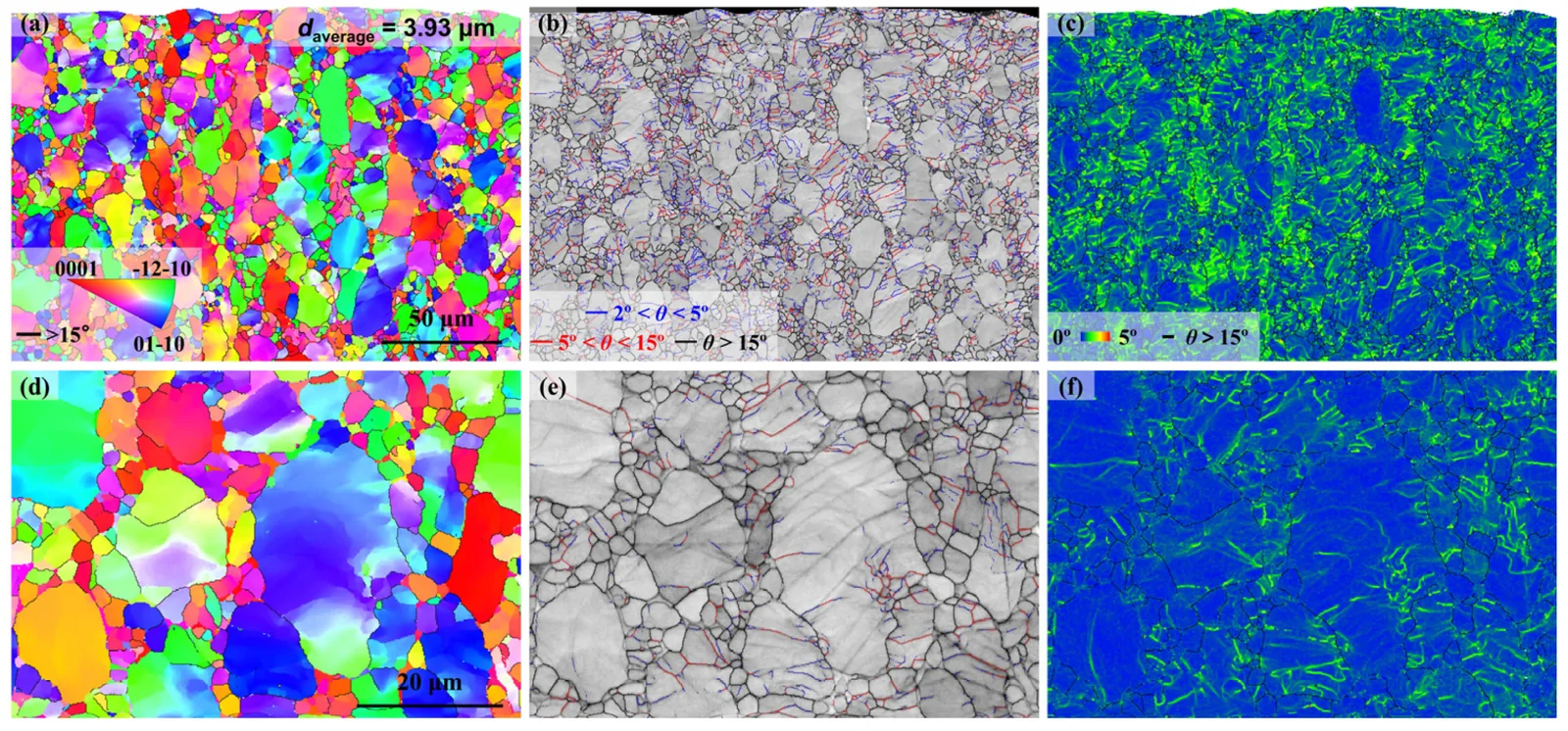
EBSD analyses performed on the superficial layer of LSP-treated Ti6Al4V alloy: (**a**–**c**) Macroscopic image; (**d**–**f**) Microscopic image; (**a**,**d**) Crystallographic orientation map; (**b**,**e**) GB map covering on band contrast map; (**c**,**f**) KAM map.

To elucidate the effect of LSP on microstructural evolution in the superficial layer, detailed EBSD analyses at both low and high magnifications were performed on the region adjacent to the top surface of the LSP-treated Ti6Al4V alloy, as shown in Figure 2. As shown in Figure 3a, the superficial layer of the Ti-6Al-4V alloy also exhibits a dual-size grain structure after LSP processing. Unlike the AR state, the coarse grains in the LSP-treated state are transformed from an elongated morphology to a relatively equiaxed shape, with sizes mainly ranging from 10 to 15 μm, which is significantly reduced. However, the size of ultrafine grains increases slightly, as shown in Figure 3d. Consequently, the difference in grain size between coarse and ultrafine grains decreases, while the coarsened ultrafine grains, which constitute a high fraction, lead to a relative increase in the average grain size. This distinctive microstructural evolution can be attributed to the differing effects of LSP on coarse and ultrafine grains. On the one hand, the coarse grains could be refined through dynamic recrystallization (DRX) induced by LSP. Whereas, since the high-fraction ultrafine grains smaller than 3 μm and ultrahigh strain rate of LSP, which render ultrashort time for microstructure evolution, the ultrafine grains with similar orientations, clustered in local regions, are merged into larger ones during simultaneous crystal tilting under LSP deformation rather than further refined. The underlying mechanism has been investigated in depth in our previous work [22]. Compared with the AR state, this reduction in grain-size gap could alleviate strain concentration at the interfaces between coarse and ultrafine grains under external stress [23]. In addition, similar to the AR state, LAGBs preferentially distribute in the rim regions of coarse grains, where high KAM values are also observed, collectively indicating a high density of GNDs in these areas [24], as shown in Figure 3b,c. However, such microstructural characteristics may lead to strain localization under external stress while simultaneously increasing resistance to deformation. Moreover, the microscopic images further reveal the specific characteristics of the LSP-treated microstructure, including equiaxed coarse grains and the distribution of LAGBs and GNDs. In addition, some fine grains also contain numerous LAGBs, confirming the merging of ultrafine grains.

**Figure 4:**
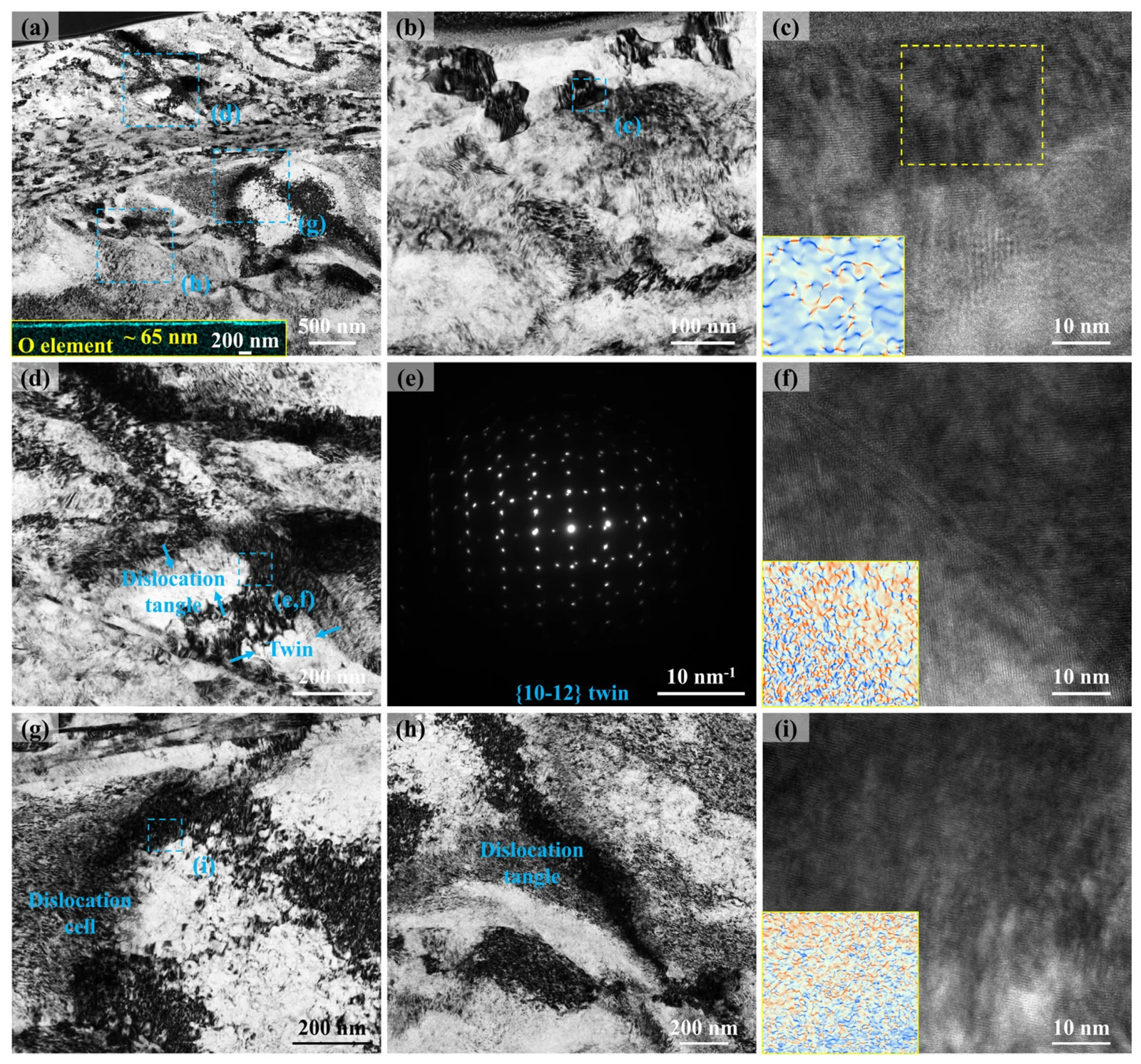
TEM analyses performed on the top region of LSP treated Ti6Al4V alloy: (**a**) Bright-field image showing the overall morphology of top region; (**b**) Bright-field image showing the nano grains in the region adjacent to surface; (**c**) HRTEM image showing the atomic structure of nano grain (Inset image showing the corresponding ε_xy_ GPA strain image of selected area); (**d**) Bright-field image showing the twin structure and surrounding dislocation structures; (**e**) SAED pattern of the twin; (**f**) HRTEM image showing the atomic structure of twin (Inset image showing the corresponding ε_xy_ GPA strain image); (**g**,**h**) Bright-field image showing the dislocation structure in inner region, and the corresponding HRTEM image showing the atomic structure (**i**) (Inset image showing the corresponding ε_xy_ GPA strain image).

To further reveal the crystal evolution caused by LSP, the TEM observations were performed on the outermost region of LSP state to demonstrate the microstructures, including grain structure, crystal defects, and interface state, in the greatest influencing area. As shown in Figure 3a, the top region of LSP state exhibits a multilayer morphology, and the nano grains dominate the outermost layer, while the grains in the inner layer exhibit obviously coarser size. Moreover, the GPA strain analysis on the nano grains in outer region reveals the relatively dense dislocations inside, as shown in Figure 4b,c. In the meantime, some {10–12} twins with large length-to-width ratio could be also observed in the outermost layer, accompanying with dislocation-tangle morphologies in surrounding region, as shown in Figure 4d,e. Moreover, high-density dislocations appear in these twins as well, as shown in Figure 4d,f. While for the inner layer, lots of dislocations tangle in the brim region of coarser grains, which exhibit the dislocation cell morphology, indicating the high dislocation density, as shown in Figure 4g,i. Moreover, dislocation walls could be commonly observed in these coarse grains as well, as shown in Figure 4h. On the other hand, for the LSP state, the thickness of oxide layer on surface is about 65 nm, as shown in Figure 4a.

### 3.2. Evolution of Superficial Microstructure and Outermost Crystal Defects Induced by Thermal Exposure

**Figure 5:**
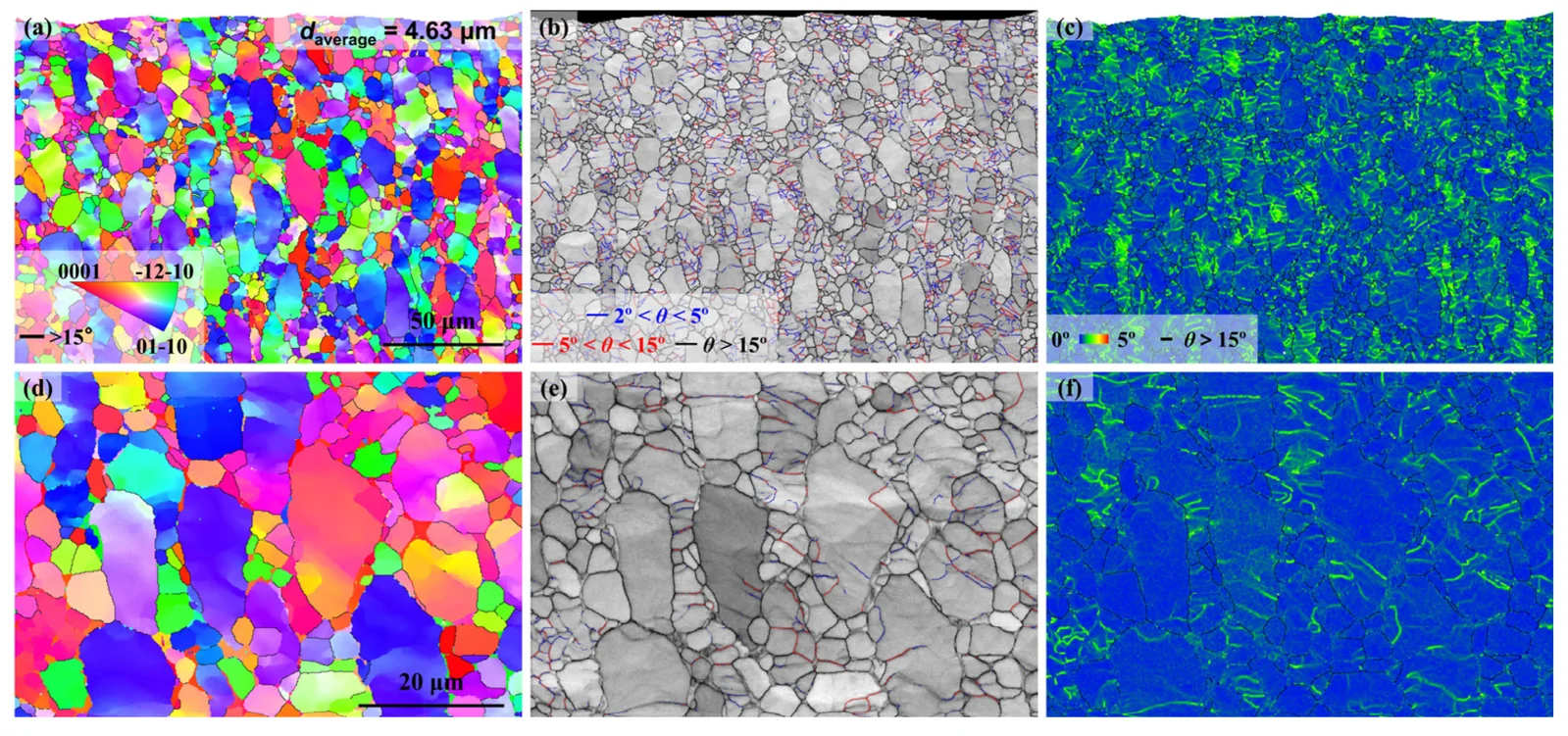
EBSD analyses performed on the superficial layer of LSP-TE-treated Ti6Al4V alloy: (**a**–**c**) Macroscopic image; (**d**–**f**) Microscopic image; (**a**,**d**) Crystallographic orientation map; (**b**,**e**) GB map covering on band contrast map; (**c**,**f**) KAM map.

Based on the LSP-processed Ti6Al4V alloy, the microstructure evolution in the superficial layer induced by thermal exposure is shown in Figure 5. It can be found that the dual-size grain structure still persists in the LSP-TE state, while the difference in grain size between coarse and fine grains is further reduced. Specifically, the size of coarse grains decreases slightly, whereas the fraction of grains ranging from 5 to 10 μm increases, and that of grains smaller than 3 μm decreases, as shown in Figure 5a,d. Consequently, the overall grain structure in the superficial layer exhibits a gradient distribution of grain size within local regions, which could be more rational than the typical dual-size structure. It can be inferred that this grain structure could contribute to strain dispersing under external deformation. Moreover, unlike the AR and LSP states, the LAGBs in the LSP-TE state preferentially distribute in finer grains rather than coarse grains, as shown in Figure 5b,e. Meanwhile, high misorientation values are also observed in these finer grains, as shown in Figure 5c,f. Combined with the distribution of LAGBs, these indicate that the fine grains in the superficial layer of the LSP-TE state possess a much higher GND density than the coarse grains, which could provide a greater ability to resist deformation and crack propagation. Additionally, the coarse grains with low GND density can benefit strain coordination capability, thereby improving the toughness of the material.

**Figure 6:**
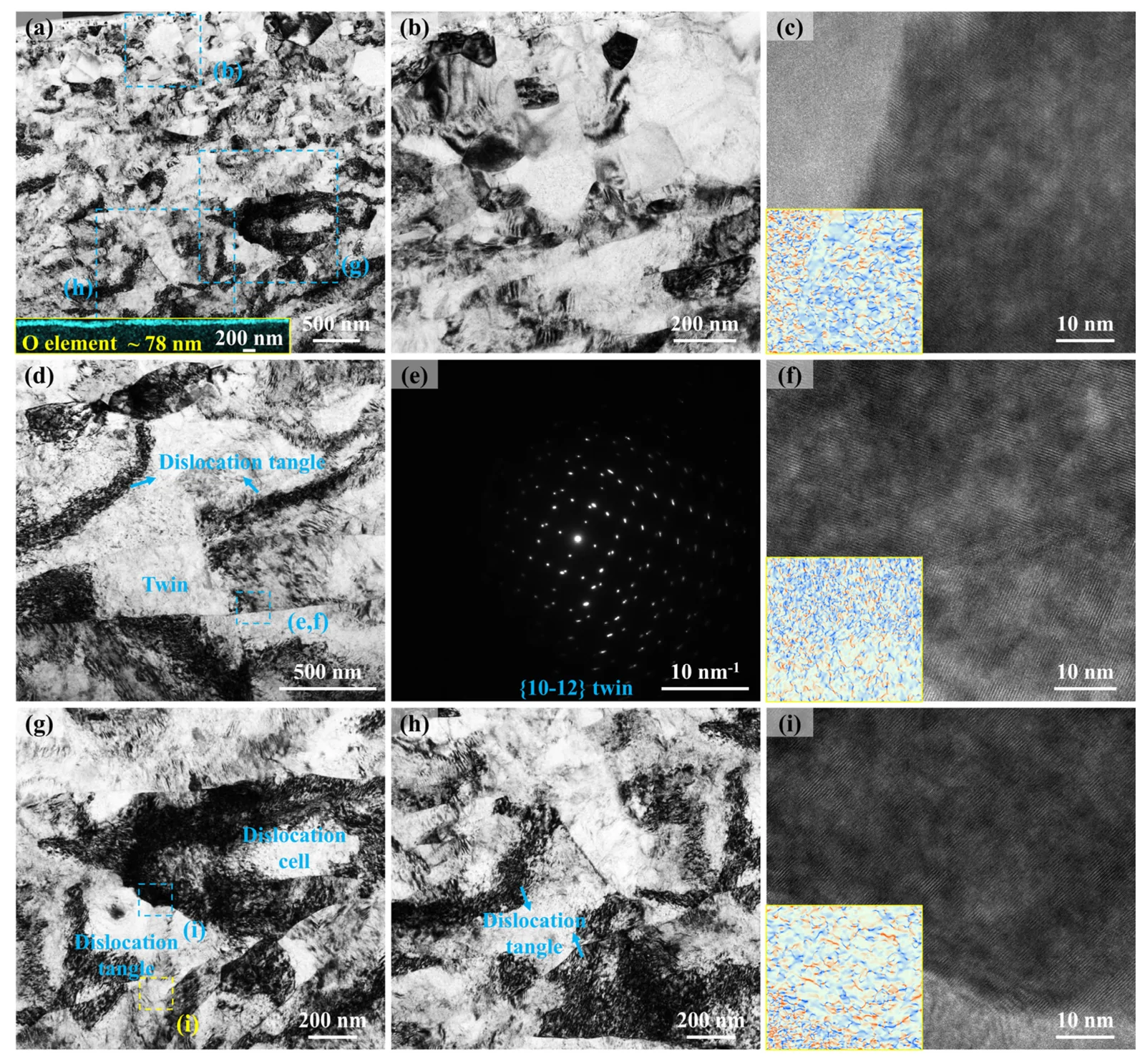
TEM analyses performed on the top region of LSP-TE-treated Ti6Al4V alloy: (**a**) Bright-field image showing the overall morphology of top region; (**b**) Bright-field image showing the nano grains in the region adjacent to surface; (**c**) HRTEM image showing the atomic structure of nano grain (Inset image showing the corresponding ε_xy_ GPA strain image); (**d**) Bright-field image showing the twin structure and surrounding dislocation structures in the deep region of this TEM specimen; (**e**) SAED pattern of the twin; (**f**) HRTEM image showing the atomic structure of twin (Inset image showing the corresponding ε_xy_ GPA strain image); (**g**,**h**) Bright-field image showing the dislocation structure in inner region, and the corresponding HRTEM image showing the atomic structure (**i**) (Inset image showing the corresponding ε_xy_ GPA strain image).

The further observations on outermost surface layer are displayed in Figure 6. As shown in Figure 6a, the multilayer grain structure of LSP state is transformed into the gradient grain structure with relatively uniform increase of grain size after thermal exposure. As shown in Figure 6b, it is noteworthy that the nano grains nearing surface are still preserved after high-temperature exposure, demonstrating the high stability of nano grains produced by LSP. Moreover, these nano grains also possess high-density dislocations inside, as shown in Figure 6c. Besides the outermost-layer nano grains, the grains in deeper layer are further refined after thermal exposure rather than coarsening, and some twins are preserved as well among these coarser grains, as shown in Figure 6d. Similar to the LSP state, {10–12} twins are the main type of these twins, as shown in Figure 6e. The preserved intensive dislocations inside twins denote their stable structure in high-temperature environment, as shown in Figure 6f. Additionally, the dislocation tangle and dislocation cell structures also appear in the sub-outer layer of LSP-TE state. Meanwhile, the grain size is decreased, as shown in Figure 6g,f. However, the dislocation density of such coarser grains is relatively lower than that of nano grains, as shown in Figure 6i. It can be deduced that the migration and decomposition of GB, cooperating with the dislocation movement, lead to these evolutions of grain and dislocation structures, which keeps consistent with the EBSD results. On the other hand, the thickness of oxide layer is slightly increased to about 78 nm after thermal exposure, indicating the great inhibition effect of dense oxide layer on element diffusion, as shown in Figure 6a.

### 3.3. Fatigue and Surface Hardness Performance

**Figure 7:**
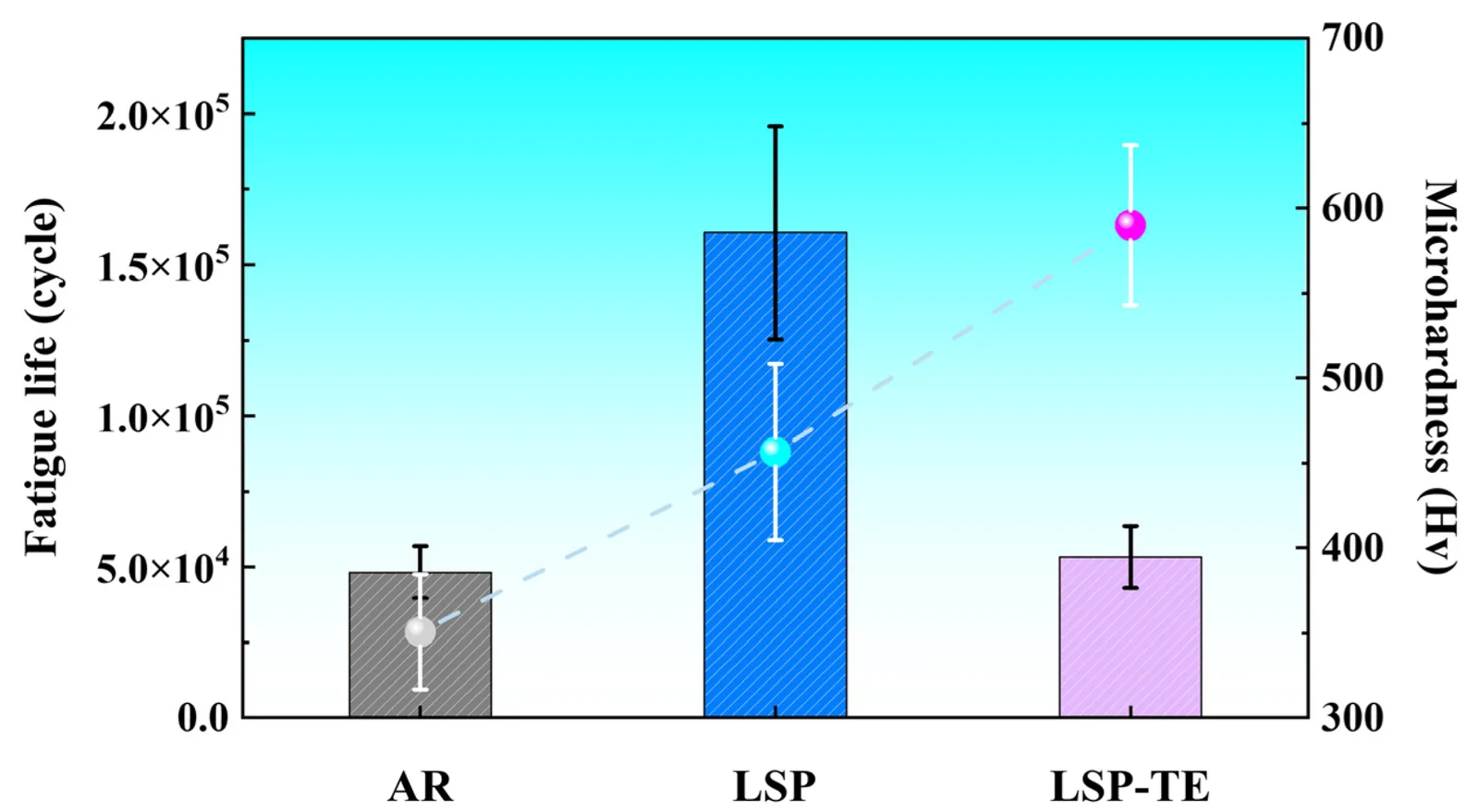
High-cycle fatigue life and surface hardness of the Ti6Al4V alloy with different states.

Besides the microstructure evolution, the high-cycle fatigue test and fracture analysis were also performed to investigate the influence of thermal exposure on fatigue performance of LSP-processed Ti6Al4V alloy, as shown in Figure 7. For the AR state, the average fatigue life under 540 MPa peak stress is about 48,224 cycles, which is similar to the reported results in previous research [25]. After LSP treatment, the average fatigue life is significantly increased to about 160,586 cycles, which is 3.33 times of AR state. Meanwhile, for the LSP-TE state, the average fatigue life is decreased to about 53,301 cycles. This value is just 10.5% higher than AR state, revealing the noticeable decrease of fatigue performance after thermal exposure.

**Figure 8:**
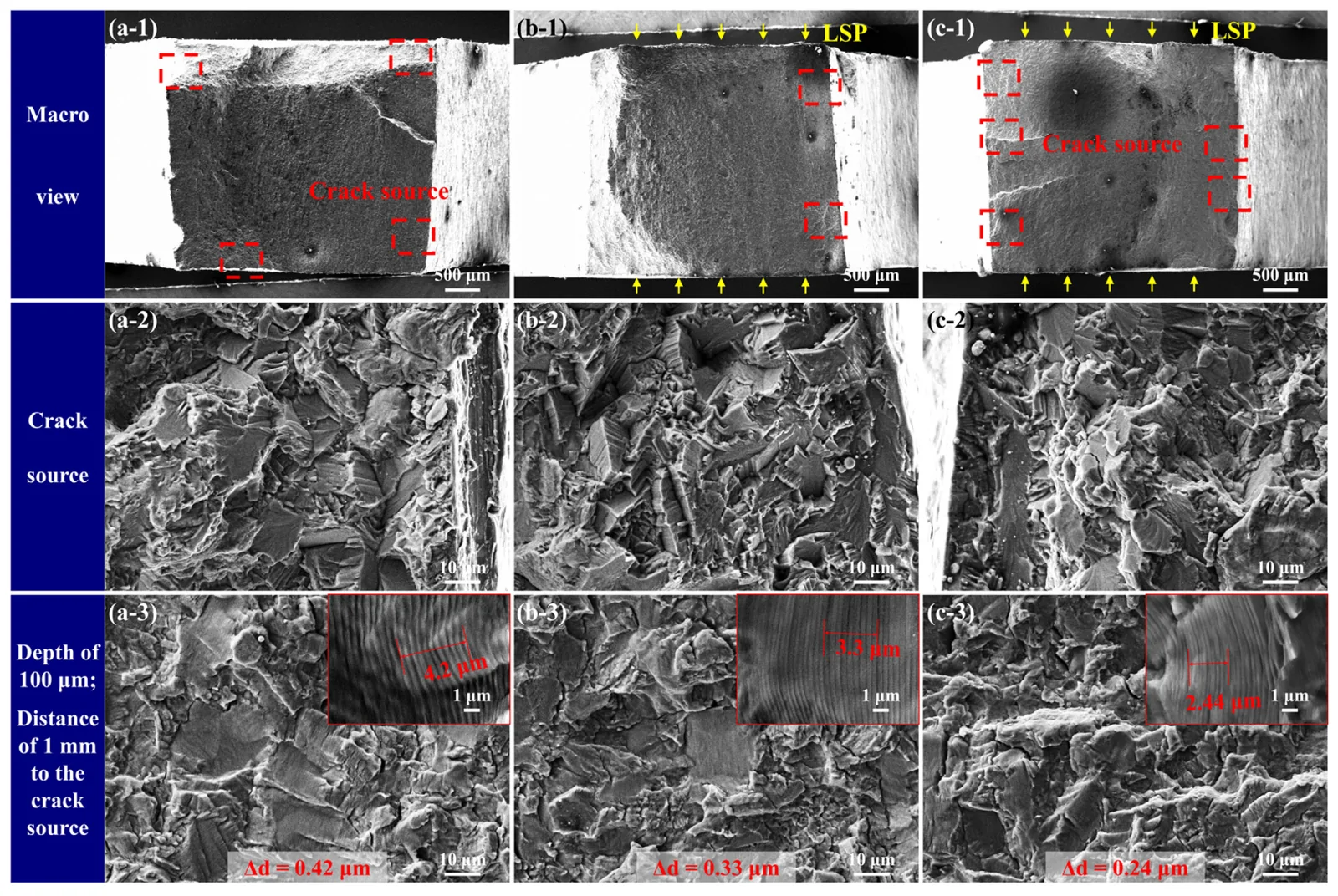
SEM observations on the fatigue fracture surface of each state: (**a**) AR state; (**b**) LSP state; (**c**) LSP-TE state; (**a-1**,**b-1**,**c-1**) Macroscopic image showing the overall morphology of fracture; (**a-2**,**b-2**,**c-2**) Microscopic image showing the detailed morphology on crack source; (**a-3**,**b-3**,**c-3**) Microscopic image showing the fatigue striation morphology in the typical region with depth of 100 μm and horizontal distance of 1 mm to crack source.

For further demonstrating the influence of LSP and thermal exposure on fatigue behavior, the fracture surface of the typical sample of each state was observed in detail. Moreover, in order to compare the inhibiting effect of microstructure on crack propagation, the fatigue striation morphology, obtained from the area in depth of 100 μm and horizontal distance of 1 mm to crack source, was utilized to calculate the average striation width. As shown in Figure 8(a-1), the fatigue crack source exhibits multiple characteristics on the surface and side wall, and some typical sources are signed in the image. The region adjacent to crack source demonstrates the relatively flat morphology, while the remote area exhibits the undulating morphology, indicating the inhibition on crack propagation, as shown in Figure 8(a-2). Figure 8(a-3) demonstrates the typical fatigue striation morphology, and the average striation width is about 0.43 μm. After LSP processing, the number of crack sources obviously decreased, which all originate from the side wall with the depth over 700 μm, as shown in Figure 8(b-1). Moreover, the crack source morphology becomes more uneven, indicating the greater inhibition on the initial crack propagation, as shown in Figure 8(b-2). According to the fatigue striation morphology in the typical region, the average striation width is about 0.33 μm, which is obviously decreased by comparing it to the AR state, as shown in Figure 8(b-3). For the LSP specimen after thermal exposure, the multiple-sites crack sources could be observed again, but all locate in the side wall with relatively large depth, as shown in Figure 8(c-1). Moreover, the initial source area of crack demonstrates flat morphology, which is similar to the AR state, as shown in Figure 8(c-2). However, the average striation width in the depth of 100 μm from the LSP-processed surface is just about 0.24 μm, which is even shorter than the LSP state, as shown in Figure 8(c-3). On this basis, it can be deduced that the outer layer of LSP-treated region could demonstrate higher fatigue resistance after thermal exposure [26], but the resistance in matrix and adjacent region influenced by LSP could be weakened by the high temperature environment, leading to the easily initiated cracks. Correspondingly, the overall fatigue life of LSP-TE state is significantly decreased. Such viewpoint could be further confirmed by the surface hardness results. As shown in Figure 7, the surface hardness of AR state is about 350.7 Hv, and this value increased to about 456.5 Hv and 589.9 Hv after LSP and LSP-TE treatment, respectively. The increased surface hardness represents the greater resisting capability to the crack initiation and propagation, especially for the superficial layer of LSP-TE state [27].

### 3.4. Influence of Thermal Exposure on the Microstructure and Fatigue Performance of LSP Processed Ti6Al4V Alloy

**Figure 9:**
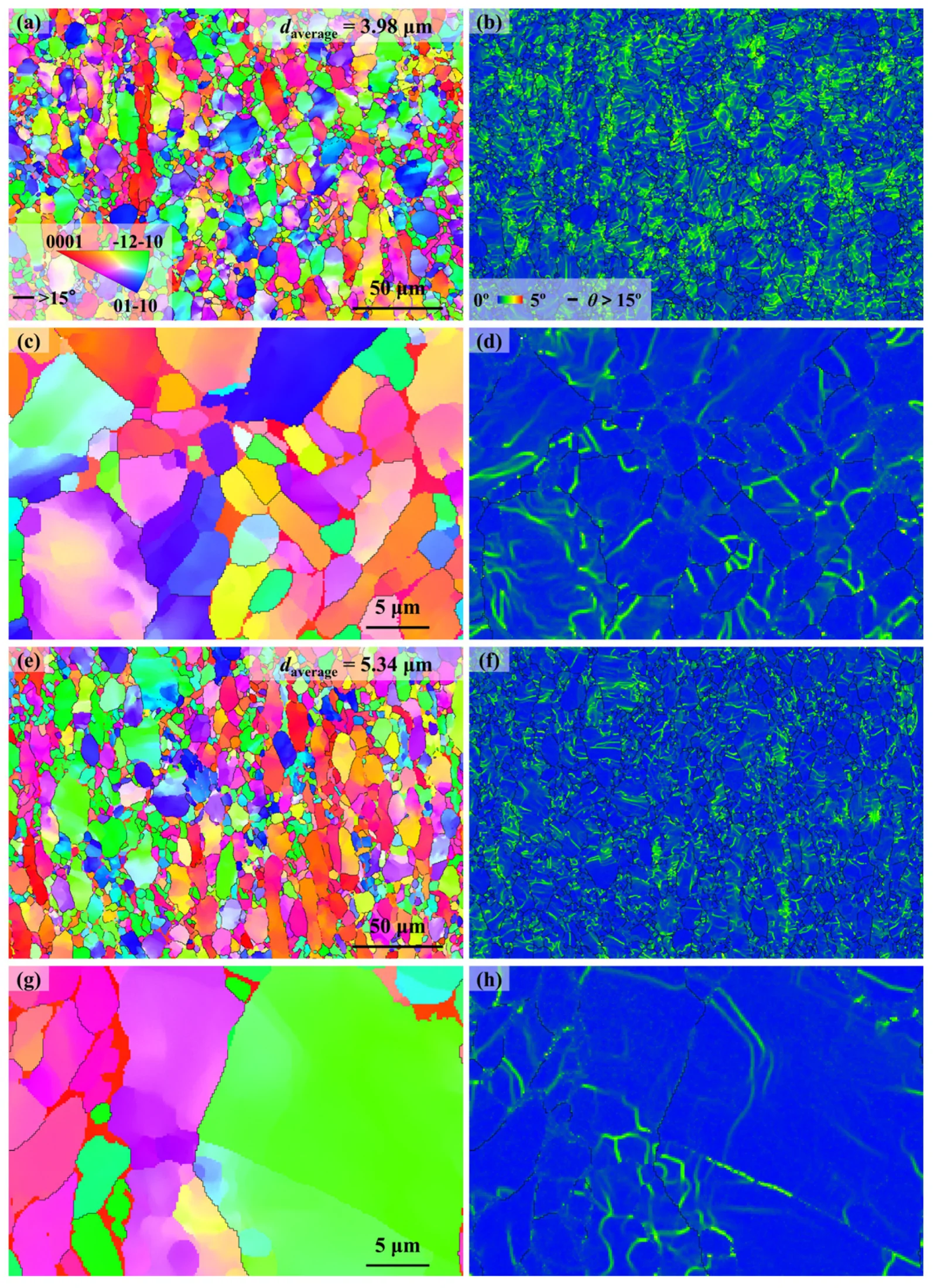
EBSD observations on the deep region of LSP- and LSP-TE-treated Ti6Al4V alloys with the depth about 1 mm: (**a**–**d**) LSP state; (**e**–**h**) LSP-TE state; (**a**,**e**) Crystallographic orientation map with macroscopic view, (**b**,**f**) corresponding KAM map; (**c**,**g**) Crystallographic orientation map with microscopic view, (**d**,**h**) corresponding KAM map.

According to the fatigue fracture morphology, especially for the fatigue striation, the LSP-treated superficial layer still preserves significant resisting capability to fatigue crack propagation, which could be even enhanced, after thermal exposure. However, the multiplied existed crack sources on the deep-layer side wall noticeably decrease the fatigue life, indicating the degradation of the nearing-matrix microstructures. In order to reveal the influence of thermal exposure on the nearing-matrix microstructure treated by LSP, the EBSD observations were performed on the area with depth about 1 mm of LSP and LSP-TE states. For the depth of 1 mm in LSP state, the microstructure evolution induced by LSP is slight, and the grain structure should lie between the superficial layer of LSP state and the matrix of AR state. As shown in Figure 9a, the dual-size grain structure can be observed, but the average grain size is relatively larger than that in the superficial layer of LSP and AR states, which could be ascribed to the residual merging effect on ultrafine grains but limited refinement on coarse grains via DRX behavior. Such deduction can be confirmed by the fine grains with sub-grains inside and the complete morphology of coarse grains without ultrafine grains in surrounding area, as shown in Figure 9c. Due to the preserved merging effect and restricted DRX on dual-size grains, the distribution of GNDs demonstrate relatively even morphology, as shown in Figure 9b,d.

As for the similar region in the LSP-TE state, the grain structure still demonstrates the dual-size feature, but it is coarser than that in the top region, accompanying with the increased average grain size of 5.34 μm, as shown in Figure 9e. This morphology can be attributed to the recrystallization behavior in the high-temperature environment, which also leads to the decreased GND density, as shown in Figure 9f. Such coarsened grain structure and reduced dislocation density would greatly decrease the resistance to crack initiation and propagation. Moreover, the detailed observations on the interface between coarse and fine grains reveal that the residual GNDs mainly distribute in the brim area of coarse grains, which is different from the top region of LSP-TE state, as shown in Figure 9g,h. This dislocation distribution infers the simultaneous weakening effects of decreased strength and strain concentration on interface, which benefit the initiation of fatigue crack as well.

**Figure 10:**
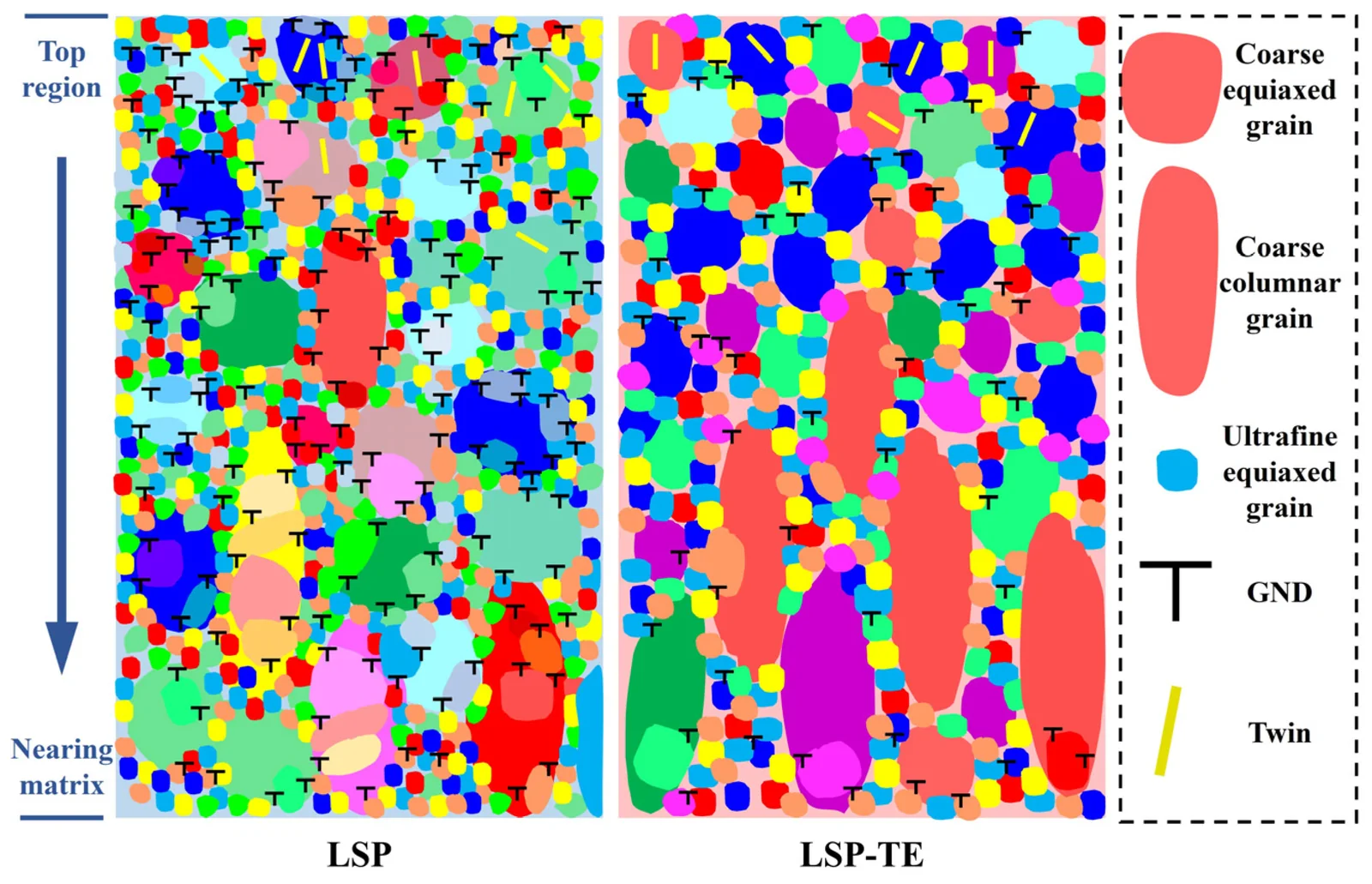
Schematic diagram showing the microstructure evolution on the influencing region of LSP induced by thermal exposure.

According to the previous research [28,29,30], grain morphology and crystal defects play vital roles in fatigue performance, which mutually determine the resistance to crack initiation and propagation. Moreover, the rational matching between grain structure and crystal defects is also a deciding factor for strain coordination in local regions under external stress [23,31]. For the Ti6Al4V alloy treated by LSP, the transiently significant deformation in superficial layer could introduce high-density dislocations and reconstruct grain structure. Based on our previous investigation, the originally coarse grains can be refined by the DRX effect of LSP. Especially for the initial columnar grains containing numerous substructures, the interaction between pre-existing LAGBs and introduced dislocations promotes the formation of high-angle grain boundaries (HAGBs), which divides the columnar grains into several equiaxed grains [32,33]. However, the ultrahigh strain rate of LSP limits the responding time for microstructure evolution, thereby restricting the degree of refinement. Moreover, since ultrafine grains with similar orientations are clustered in local areas of the AR state, the transient deformation preferentially forces crystal tilting of these grains, leading to a decrease in orientation difference, which merges the ultrafine grains into larger ones containing some LAGBs. Consequently, the difference in grain size is obviously reduced after LSP treatment. In addition, the transiently severe deformation on surface also introduces some twins into the coarser grains in superficial layer. With the depth increased, the influence of surface deformation is gradually decreased, resulting in the disappearance of twins, and the weakened multiplication and movement of dislocations could impair the DRX effect. Accordingly, more columnar grains re-emerge in the deeper region of LSP state, and their fraction is increased with the increase of depth. Moreover, the weakened deformation also affects the merging behavior of ultrafine grains, while the superposition of more shock waves could relatively make up the driving force for crystal tilting, leading to the remaining grain-merging phenomenon. Consequently, the density of residual dislocations in the deep region, particularly for the GNDs, is lower than the top region, and the dislocation distribution could be more uniform due to the decreased substructures in coarse grains and remaining merging grains. After thermal exposure, the input thermal energy accelerates the GB migration and recrystallization. The ultrafine grains are slightly coarsened due to the GB migration outwards, while the recrystallization on coarse grains transforms the substructures into new grains. Especially in the outermost region, the coarser grains with lots of substructures inside, formed by the transient deformation of LSP, are refined into ultrafine ones, which even decreases the average grain size in such layer. Hence, the difference in grain size between dual-size grains is further decreased, and more dislocations distribute in the finer grains since the decomposition of initial GB [34,35]. Moreover, due to the ultrahigh strain rate of LSP, the high-density dislocations around twins could preserve the twin structure under the thermal influence, meaning lots of twins remained inside the coarser grains in outermost layer. With the increase of depth, the influence of LSP on grain structure and crystal defects is gradually decreased, and the crystal tilting and GB migration induced by thermal exposure further promote the re-generation of coarse columnar grains. Moreover, the GB migration of fine grains further increases the size of equiaxed grains, but the densely present coarse grains restrict their outwards GB migration, limiting the dislocation generation inside. Moreover, the simultaneous recrystallization effect inhibits the formation of substructures in both fine and coarse grains. Comparatively, some substructures could be preserved from the coarse grains of LSP state, remaining intensive GNDs in the brim region. The related microstructure evolutions are demonstrated in Figure 10.

The microstructural evolution induced by LSP and subsequent thermal exposure fundamentally governs the fatigue behavior of the Ti6Al4V alloy, as reflected by the fatigue life and crack propagation characteristics. For the AR state, the dual-size grain structure with high-density dislocations accumulated in the coarse columnar grains (Figure 1) provides considerable resistance to deformation at the initial stage. However, the columnar morphology and high dislocation density within these coarse grains weaken the strain accommodation capability, particularly near GBs where excessive dislocation accumulation occurs. The strong crystallographic anisotropy of columnar grains, together with their limited slip systems, further exacerbates strain localization and promotes crack initiation under cyclic loading, resulting in a relatively insufficient fatigue life and a larger fatigue striation width [36]. After LSP treatment, the superficial microstructure is significantly reconstructed. The refinement of coarse grains via DRX, the merging of ultrafine grains, the transformation of columnar grains towards equiaxed grains, and the introduction of high-density dislocations and twins collectively produce a more homogeneous superficial layer with a reduced grain size gap. This microstructural state enhances the resistance to crack initiation by dispersing strain and suppressing dislocation accumulation at GB of coarse grains. Meanwhile, the high-density dislocations and reconstructed grains effectively inhibit crack propagation, as evidenced by the significantly reduced fatigue striation width and the decreased number of crack initiation sites, which all originate from the side wall at depths exceeding 700 μm. Consequently, the fatigue life of the LSP state is increased by more than two-fold compared to the AR state, demonstrating the pronounced strengthening effect of LSP on fatigue performance. However, after thermal exposure, the fatigue performance of the LSP-TE state deteriorates significantly. Interestingly, the fatigue striation width in the superficial layer of the LSP-TE state is further reduced, compared to the LSP state, indicating that the outermost layer of the LSP-treated region still exhibits enhanced resistance to crack propagation after thermal exposure. This can be attributed to the rationally distributed grain structures with different sizes, accompanying with the low dislocation density of coarse grains and high dislocation density of fine grains. Such a microstructure could simultaneously benefit the high strength and toughness while improving the strain coordination among different-sizes grains. Nevertheless, for the multilateral structure such as turbocharged components, the double-sided LSP could only influence part of the region, and the matrix and adjacent region nearing side walls also play a critical role in fatigue performance, where the thermal exposure induces significant microstructural degradation. In particular, the re-emergence and coarsening of columnar grains in the matrix region, which exhibit strong crystallographic anisotropy and limited deformation compatibility, further exacerbate strain localization and provide preferential paths for crack initiation and propagation. The coarsened grains and excessive dislocation distribution in local region also weaken the strain accommodation capability and reduce the resistance to crack initiation, leading to the emergence of multiple crack initiation sites. These cracks initiate preferentially in the weakened matrix region and propagate rapidly, ultimately causing the significant reduction in fatigue life. Therefore, the fatigue performance of the LSP-TE state is governed by the competition between the preserved strengthening effect in the outermost layer and the thermal degradation in the matrix region, with the latter playing a dominant role in determining the overall fatigue life.

## 4. Conclusions

In the present research, the influence of thermal exposure on the microstructure and fatigue performance of LSP-treated Ti6Al4V alloy with double-side strengthened surfaces was systematically investigated. The main conclusions can be summarized as follows:

(1) LSP refines the coarse grains through DRX and merges the clustered ultrafine grains via crystal tilting, thereby reducing the grain size difference in the superficial layer. Specifically, the columnar grains are transformed into the equiaxed morphology after LSP. Moreover, high-density dislocations and {10–12} twins are also introduced into superficial layer. However, the GNDs still preferentially distribute in the brim region of coarse grains, which is similar to the AR state. While in the deep region nearing the matrix, the insufficient DRX effect leads to the re-appearance of columnar grains, but the merged grains benefit the relatively homogeneous distribution of GNDs.

(2) After thermal exposure, the dual-size grain structure is retained in the superficial layer, but the grain size difference is further reduced, and the grains with different sizes rationally distribute in local region. The nanograins and twins remain stable in the outermost layer, while the finer grains in the superficial layer possessed higher GND density than the coarse grains. However, the nearing-matrix region undergoes grain coarsening and dislocation recovery, cooperating with the accumulation of GNDs in brim area of coarse grains, and the columnar grains re-generate in such a region, leading to a degraded microstructure with reduced resistance to crack initiation.

(3) LSP significantly increased the fatigue life to 2.33 times that of the AR state, with a reduced fatigue striation width and fewer crack initiation sites. After thermal exposure, the fatigue life decreased to only 10.5% above that of the AR state, despite the superficial layer exhibiting an even smaller fatigue striation width. This degradation was attributed to the multiple crack initiation sites in the thermally degraded matrix and adjacent regions.

## Data Availability

The original contributions presented in this study are included in the article. Further inquiries can be directed to the corresponding author.
